# Outcomes of usual chiropractic, harm & efficacy, the ouch study: study protocol for a randomized controlled trial

**DOI:** 10.1186/1745-6215-12-235

**Published:** 2011-10-31

**Authors:** Bruce F Walker, Barrett Losco, Brenton R Clarke, Jeff Hebert, Simon French, Norman J Stomski

**Affiliations:** 1 School of Chiropractic and Sports Science, Murdoch University, Perth, Australia; 2 School of Chemical and Mathematics and Sciences, Murdoch University, Perth, Australia; 3 Primary Care Research Unit, Melbourne University, Melbourne, Victoria, Australia; 4 School of Public Health and Preventive Medicine, Monash University, Melbourne, Victoria, Australia

## Abstract

**Background:**

Previous studies have demonstrated that adverse events occur during chiropractic treatment. However, because of these studies design we do not know the frequency and extent of these events when compared to sham treatment. The principal aims of this study are to establish the frequency and severity of adverse effects from short term usual chiropractic treatment of the spine when compared to a sham treatment group. The secondary aim of this study is to establish the efficacy of usual short term chiropractic care for spinal pain when compared to a sham intervention.

**Methods:**

One hundred and eighty participants will be randomly allocated to either usual chiropractic care or a sham intervention group. To be considered for inclusion the participants must have experienced non-specific spinal pain for at least one week. The study will be conducted at the clinics of registered chiropractors in Western Australia. Participants in each group will receive two treatments at intervals no less than one week. For the usual chiropractic care group, the selection of therapeutic techniques will be left to the chiropractors' discretion. For the sham intervention group, de-tuned ultrasound and de-tuned activator treatment will be applied by the chiropractors to the regions where spinal pain is experienced. Adverse events will be assessed two days after each appointment using a questionnaire developed for this study. The efficacy of short term chiropractic care for spinal pain will be examined at two week follow-up by assessing pain, physical function, minimum acceptable outcome, and satisfaction with care, with the use of the following outcome measures: Numerical Rating Scale, Functional Rating Index, Neck Disability Index, Minimum Acceptable Outcome Questionnaire, Oswestry Disability Index, and a global measure of treatment satisfaction. The statistician, outcome assessor, and participants will be blinded to treatment allocation.

**Trial registration:**

Australia and New Zealand Clinical Trials Register (ANZCTR): ACTRN12611000542998

## Background

The success of any therapy is often based on the questions "Is it safe? Is it effective?". For chiropractic therapy there has been some research to ascertain effectiveness [1] and also for spinal manipulation [2] but there has been very little to document its safety. The safety profile of any therapy is a cornerstone of modern practice as it provides protection for the public and informs the consumer consent process. Trials of chiropractic therapy have shown mixed results which, together with its current use for multiple indications, make research into aspects of its safety important.

There are several published reports about the safety of chiropractic treatment. A single arm study conducted by Senstad et al in 1996 [3] reviewed the type, frequency, and characteristics of unpleasant side effects after spinal manipulation. About half of the patients reported at least one adverse event some time during the course of a maximum of six treatments. Of these, the most common adverse events were local discomfort (53%), headache (12%), tiredness (11%), or radiating discomfort (10%). Adverse events were mild or moderate in 85% of patients. Limitations of this study included that the chiropractors gathered their own data; responders could not be compared with non-responders; and reports could not be obtained from patients who attended one treatment only. It is possible that some opted out because of unpleasant reactions, potentially making the results an underestimate.

Hurwitz et al compared the rate of adverse events between two randomly allocated groups of neck patients who received either manipulation or mobilisation from chiropractors [4]. About a third of all participants reported at least one adverse event. The most common adverse events were increased pain (27%), headache (15%), tiredness (12%), radiating pain (6%), and dizziness (4%). Participants who received manipulation were more likely to experience an adverse event than participants who received mobilisation.

A prospective survey of chiropractors in the United Kingdom examined adverse events following manipulation of the neck [5]. No serious adverse events were reported. Of the adverse events reported, the most notable were increased neck pain (7%) and increased shoulder/arm pain (4%), muscle stiffness and headache (4%). A potential limitation of this study was that data were collected by the chiropractors administering the intervention, which may have led to an underestimation of adverse events.

The fourth study was a prospective study of patients who received chiropractic care for neck pain [6]. Just over half of the participants experienced at least one adverse event following any of the three treatments, and 13% of the adverse events were severe in intensity. About 75% of all reported adverse events were musculoskeletal or pain related. Other less common adverse events included tiredness, dizziness, nausea, or ringing in the ears.

The main limitation associated with these four studies above [3-6] was that three of the studies were uncontrolled. These uncontrolled studies may have overestimated the frequency of adverse events because the reported events may be associated with either natural history [6] or a placebo response [7].

Another limitation concerns the involvement of chiropractors in collecting information about adverse events directly from the participants in two of the studies [3,5]. The involvement of chiropractors may have led to an underestimation of adverse events as the patients may have been reluctant to disclose the events they experienced.

Given the limitations associated with previous studies of adverse events during chiropractic care [3-6], it is evident that controlled studies are warranted to develop a better understanding of the rate of adverse events in chiropractic care. This proposition reflects the findings of a recent systematic review [8] that concluded there were no robust data concerning rate of adverse events after chiropractic treatment and recommended urgent investigations to provide definitive conclusions as to its safety profile.

In summary, the literature reveals that adverse events do happen from chiropractic therapies. However, previous studies have limitations and therefore we do not know the frequency and extent of these events particularly when compared to sham treatment.

## Aims

1. To establish the frequency and severity of adverse events from usual short term chiropractic treatment of the spine when compared to a sham treatment.

2. To establish the efficacy of usual short term chiropractic care for spinal pain when compared to a sham intervention.

## Materials and methods

This study will be a randomised, placebo-controlled trial comparing usual short term chiropractic care and a sham intervention of no known benefit for participants with non-specific spinal pain of more than one week in duration. The inclusion of a placebo group will allow for a comparison of adverse events between active chiropractic care and those that are a manifestation of either "sham" treatment or natural history. Participants will be randomly allocated to receive usual chiropractic care or a sham intervention consisting of non-functional therapeutic ultrasound and a de-tuned Activator^® ^instrument [9]. The reporting of the results of this trial will accord with both the extended CONSORT statement for Randomized Trials of Nonpharmacologic Treatment [10] and the CONSORT extension for Better Reporting of Harms in Randomized Trials [11].

### Study Sample and Participant Enrolment

Participants will be recruited in Perth, Western Australia by the use of newspaper advertisements. Any person who responds to the advertisement by phone will be considered a potential candidate and will receive through the mail the following documents:

• an information letter;

• a pain diagram to assess symptom location and distribution;

• the Numerical Rating Scale (NRS) [12] on which the patients will score their current overall level of spinal pain;

• Functional Rating Index (FRI) [13];

• a questionnaire enquiring about the minimum amount of change in pain levels that potential participants would consider to be an acceptable outcome [14];

• a medical history checklist.

These documents will be returned in a reply paid envelope and the information will be used to determine eligibility. To be considered for inclusion, potential participants must be adults (18 years or older), literate in English, and have had spinal pain (neck pain, mid-back pain, or low back pain) for more than 1 week. In addition, potential participants must score at least 3 on the NRS and 12 on the FRI as this allows measurement of a minimal clinically important difference in the improvement direction [15].

Potential participants will be excluded if they:

• have been previously unable to tolerate common chiropractic treatments, for example: manipulation; mobilisation; Activator^®^; flexion distraction therapy; traction; soft tissue massage; trigger point therapy; sacro-occipital technique; ultrasound; interferential therapy; and Transcutaneous Electrical Nerve Stimulation (TENS).

• have any of the following conditions: spinal pain related to cancer or infection; clinically important fracture of the spine; spondyloarthropathy; known osteoporosis; progressive upper or lower limb weakness; symptoms of cauda equina syndrome or other important neurological condition; recent disc herniation; cardiovascular disease likely to be a contraindication to spinal manipulation; uncontrolled hypertension; cognitive impairment; blood coagulation disorder; spinal surgery in the last year; previous history of stroke or transient ischaemic attacks; have a pacemaker or other electrical device implanted; are currently pregnant;

• have a current compensation claim;

• have a substance abuse problem;

• cannot commit to study protocol.

Potential participants who satisfy the inclusion criteria will be sent a more detailed information letter. Two days later, they will be contacted by phone to determine if they are interested in participating, and if so an appointment will be made to attend Murdoch University.

### Chiropractor Participants

The study interventions will be administered at the clinics of registered chiropractors in Perth, Western Australia. Recruitment of about 12 chiropractors will be by way of open advertisement to the profession using contact details in the public domain or from the online Register of Chiropractors. To be eligible, chiropractors must have had at least two years of clinical experience and regularly use spinal manipulation in the mix of their therapy modalities. Chiropractors who administer the sham intervention must have both ultrasound and Activator^® ^instruments available.

### Treatment allocation

When participants visit Murdoch University and after baseline measures and consent have been obtained, one of the research staff, excluding the research assistant (RA) and statistician, will randomly allocate the participants to one of the two groups using a 1:1 allocation. A statistician will generate a permuted block randomization sequence allocating persons to either chiropractic care or the sham group. The group assignment will be written on small cards and placed in opaque envelopes.

For ethical reasons, participants in the sham group will be offered two treatments of usual chiropractic care two weeks after receiving the last sham treatment and following receipt of their final outcome measures by the RA. Any participant who discontinues treatment will be followed-up to determine the reason for discontinuation.

### Pilot Study

A pilot study, using the first five participants recruited, will be undertaken to evaluate process and resource issues. In particular, we will examine whether the participants were able to complete the questionnaires in full without difficulty and the length of time required. All research team members involved with baseline assessments and randomisation will meet after the first five participants have been randomised to reach a consensus decision on the feasibility of administering the questionnaires. In the absence of explicit recommendations about the required sample size to examine feasibility issues, we determined that five participants would be sufficient [16].

### Intervention/treatment

Chiropractors will be asked to deliver one of the two study interventions. The study interventions are as follows:

1. Short term usual chiropractic care: a series of two chiropractic treatments to the spine as deemed suitable by a registered chiropractor. Treatments include one or more of the following: spinal manipulation, mobilisation, drop piece, traction, electro- therapies, ultrasound, Activator^®^, soft tissue techniques and other manual techniques offered by the chiropractor. This treatment approach replicates a common pragmatic approach in the field [1].

2. Sham Group: participants will receive two sessions of de-tuned ultrasound and de-tuned Activator^® ^treatment by a chiropractor to the regions where spinal pain is experienced. There is no known treatment benefit from the de-tuned ultrasound machine and it has been established in previous trials as a credible treatment option by patients [17,18]. Chiropractors will be directed to wind the Activator^® ^head force to minimum and then administer the force through a wooden tongue depressor at random locations in the spine near the areas of concern. The dimensions of the depressor are 19 mm wide and 150 mm long, although on average only 600 mm of the length will be in contact with the skin. This means a surface area of 1140 mm^2^. We measured the force this will provide to the skin using a force transducer. The mean of 5 measured attempts was a very low 3.7 kPa. To increase the perceived "hands on" credibility, the chiropractor will be trained to gently place one hand on an area adjacent to the participant's spine while delivering the ultrasound and Activator^® ^therapies. The four chiropractors recruited to deliver the sham treatment will be asked to administer the sham interventions as they normally use Activator^® ^tools and ultrasound and with the same enthusiasm they would use for all other therapies.

### Withdrawing Participants from this Study

A stopping rule was defined for withdrawing participants from this study in accordance with recommendations in the CONSORT extension for Better Reporting of Harms [11]. If a participant reports to their chiropractor or research staff a severe adverse event they will be withdrawn from the study and referred to their local doctor or hospital emergency department depending on the nature of the event. Each participant will complete an adverse event questionnaire two days after each chiropractic appointment and asked to then return it in a reply paid envelope. Any adverse event will be classified as severe if it is thought to be an emergency situation e.g. stroke or the associated pain intensity is rated above 8/10 on an 11 point NRS, which accords with the definition for a serious adverse event by Rubinstein et al [6].

### Ethical Clearance

All participants (chiropractors and patients' with spinal pain) will provide voluntary written informed consent with a full understanding of what study participation entails and the potential risks. Ethics approval has been obtained from Murdoch University's Human Research Ethics Committee (Reference number 2011/109).

### Demographic Material and Assessment of Outcomes

#### 1. Chiropractor Demographics and Usual Chiropractic Care Characteristics

For the participating chiropractors, the following demographic characteristics will be collected: age, years in practice, institution from which qualifications were obtained, and speciality certification or additional qualifications. A treatment checklist will be used to collect information about the types of treatments used in each usual chiropractic care appointment. Collection of the demographic and treatment information accords with the CONSORT Recommendations for the Reporting of Nonpharmacologic Trials [10]. Information about the types of treatment used will also be used for later analysis and correlation with adverse events.

#### 2. Baseline Assessment

The following questionnaires will be administered at baseline: demographic questionnaire; NRS [12]; FRI [13]; Fear-Avoidance Behaviour Questionnaire (FABQ) [19]; SF-36 [20]; STarT Back Musculoskeletal Screening Tool [21]; and the Pain Catstrophising Scale (PCS) [22]. Also, the primary area of concern for each participant will be identified and one of the following questionnaires specific to that area will be administered: the modified Oswestry Disability Index (ODI) [23]; or Neck Disability Index (NDI)[24].

Descriptive statistics from the baseline measures including the SF-36, STarT Back Musculoskeletal Screening Tool, and demographic questionnaire will be examined to determine if clinically important differences are evident between the usual chiropractic group and sham group at baseline. The SF-36 is a well validated and extensively used measure of general health status [25]. The STarT Back Musculoskeletal Screening Tool is brief validated instrument that has been developed to identify people with chronic pain who are more likely to experience persistent symptoms [21]. The demographic questionnaire enquires about age, sex, race/ethnicity, education, household income, marital status, employment status, smoking, and alcohol and legal drug consumption. These items were derived from classifications used by the Australian Bureau of Statistics [26].

A baseline symptom checklist will be administered to examine whether the adverse events that participants reported after each chiropractic treatment were actually an adverse event or the persistence of a presenting complaint. This will be achieved through comparing data from the symptom checklist taken at baseline to data from the adverse event checklist collected after each chiropractic treatment.

The NRS contains an 11-point scale varying from 0 (no pain) to 10 (worst pain imaginable) [12]. It is a valid, reliable and responsive measure of pain intensity [27]. Participants will be asked to use the scale to rate: their worst level of pain in the last two days; average pain level in the last two days; best pain level in the last two days; and current pain.

The FRI contains 10 questions which enquire about the impact of neck and back pain on physical function. It was designed to assess conditions where more than one area of the spine is involved and is valid, reliable and responsive [13].

The NDI was designed to measure neck-specific disability. The questionnaire has 10 items concerning pain and activities of daily living. It has demonstrated validity, reliability and responsiveness [24].

The modified ODI is a region-specific outcome measure that has been shown to have high levels of reliability, validity and responsiveness in patients with low back pain [23]. It assesses impact of low back pain on physical function and activities of daily living.

The FABQ will be used to assess participant's beliefs about the relationships between back pain, work, and physical activity. The FABQ has good test-retest reliability [19], and predictive validity for future episodes of LBP [23] and clinical outcome [15].

Catastrophising will be assessed with the PCS [22]. The PCS assesses different dimensions of negative thoughts related to pain: rumination, magnification, and helplessness. It contains 13 items scored on a 5-point Likert-type scale varying from 0 ("not at all") to 4 ("all the time"). The PCS has been shown to be a reliable and valid measure which predicts postoperative pain intensity following lumbar spine surgery [28].

#### 3. Assessment of Adverse Event Events

To accord with the CONSORT extension for Better Reporting of Harms [11], it is necessary to *a priori *define anticipated and unanticipated adverse events and explicitly state these definitions in reporting the methods for this study. Anticipated adverse events for this study are defined as the adverse events listed in the questionnaire developed for this study and any of the other adverse events reported in the studies conducted by Senstad et al. [3], Hurwitz et al. [4], Rubinstein et al. [6], and Theil et al. [5]. Unanticipated adverse events will be defined as stroke, fracture, dislocation, or disc injury.

Information about adverse events will be collected after each chiropractic appointment. Participants will be asked to complete the questionnaire two days after each appointment as adverse events typically manifest within two days of chiropractic treatment [3,4,6].

The adverse events questionnaire [Additional file 1] was derived from a checklist used by Cagnie et al. [29]; open-ended questions from Senstad et al. [3] and Hurwitz et al. [4]; adverse events reported in RCT's [4,6,29-32]; and two surveys [5,33]. It consists of items which enquire about the onset and severity of adverse events, and items about the impact of adverse events on activities of daily living. We decided to use a questionnaire which consisted largely of predefined response options as previous studies have demonstrated that the use of predefined response options results in more comprehensive reporting of adverse events [34].

#### 4. Assessment at Two Week Follow-Up

We will administer at two week follow-up the NRS [12], FRI [13], and the area specific instruments modified ODI [23] or NDI [24]. These questionnaires will be used to establish the short term efficacy of brief chiropractic care for spinal pain. The FABQ [19] and PCS [22] will also be administered to examine whether cognitive aspects related to the experience of pain influence pain severity, physical function and adverse events.

Also, a global measure of treatment satisfaction [27] and global measure of perceived change [35] will be administered at two week follow-up. The global measure of treatment satisfaction [27] will be used to examine the association between adverse events and treatment satisfaction. It will be measured on a five point categorical scale (very dissatisfied = 0; dissatisfied = 1; no preference = 2; satisfied = 3; very satisfied = 4). The global measure of perceived change [35] will be used to examine the association between adverse events and overall perceived change in the main complaint. It will be measured on an 11 point continuous scale varying from -5 to +5 (-5 = much worse, 0 = unchanged, +5 much better).

Blinding will be assessed at two weeks with a questionnaire [36] that asks the participants if they were in the real treatment group or the sham treatment group. The possible responses are: real treatment group; sham group; or don't know.

### Blinding

Practitioners are unable to be blinded with respect to the participants' treatment group allocation. The statistician and research assistant will remain blind to treatment allocation. We aim to blind participants to the assigned treatment throughout the trial.

### Sample Size

The sample size was based on the number needed to detect a significant difference in the primary outcome (adverse event: yes/no) between groups. We make the assumption that in the usual chiropractic group 45% of patients will experience an adverse event and that the rate in the sham group will be 25%. We believe this is a very conservative estimate given that a) the participants in the Senstad et al. study [3] had 6 treatments and there was a rate of 55% adverse events and b) that for the sham treatment previous studies have shown a mild adverse event rate of 7% [37] using the same de-tuned ultrasound method. Therefore, using a 2-sided test and alpha level of 0.05, recruiting 90 participants in each group (180 total) will provide 80% power to detect a difference this large or larger. G*Power 3.1.3 was used to calculate the sample size [38].

#### Statistical Methods

Data will be entered and analysed in SPSS Version 17. Data will be checked for implausibility's by using a frequency analysis of each variable. In addition, a second check of half the data entered will be made in detail for errors. Descriptive statistics will be derived for the participants' and chiropractors' demographic characteristics, and the types of treatments used. The measurements taken at baseline for the participants will be compared to establish whether the groups are similar.

#### Analysis of Adverse Events

The primary outcome (adverse event; yes/no) will be compared between groups. The severity and frequency of adverse events will be reported descriptively. Logistic regression will be used to examine associations between adverse events and several factors. Factors which have been significantly associated with adverse events in previous studies will be selected for this study. These factors comprise treatment satisfaction [4], manipulation/mobilisation [4], global improvement [4], GP visit in last six months [6], duration of neck/back pain [6], medication use [29], gender [29], and age [29]. The relative risk [39] will be calculated using a two by two contingency table and 95% confidence intervals. The number needed to harm [40] will be calculated with 95% confidence intervals to identify the number of individuals needed to be exposed to a risk factor (chiropractic treatment) over a specific period to cause harm (adverse outcome) in one patient that would not otherwise have been harmed. Data will be assessed by using per protocol analysis. In addition, an intention to treat analysis will be undertaken by using sequential regression multiple imputation [41].

#### Analysis of Secondary Outcomes

The efficacy of short term usual chiropractic care will assessed in two ways. First, for the repeated measures ANOVA will be used to compare differences between groups for the pain and physical function measures. Second, the minimum acceptable outcome [14], which involves establishing what participants consider to be a "minimum acceptable outcome" for which they would undertake a procedure. The proportion of participants meeting this target in each group will be reported descriptively and the groups will be compared by using a chi-squared test [14].

Blinding to treatment allocation will be assessed by using the Bang Index (BI) [36] which will allow us to determine if a significant proportion of participants guessed that they were in opposite treatment group. A BI value of < -0.2 indicates that significant proportion guessed wrongly, BI between 0.2 and -0.2 indicates participants guessed randomly, and BI > 0.2 indicates that the study was unblinded.

### Study Flow

**Figure 1:**
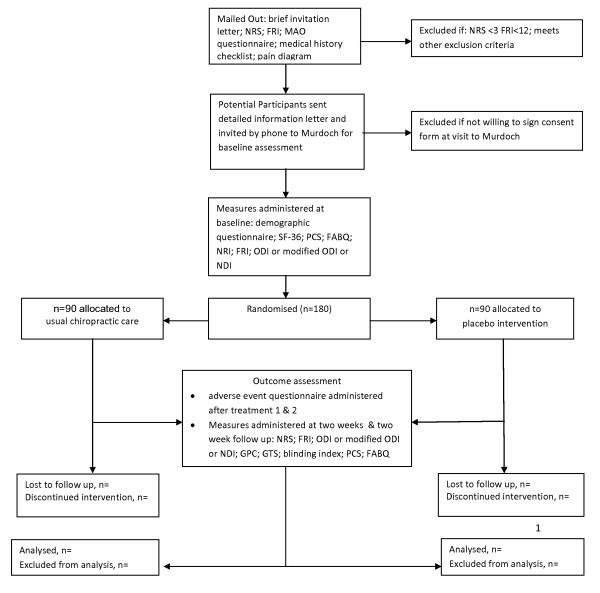
**Study Flow**.

A flow chart of the study is seen in Figure 1.

## Discussion

To our knowledge, this is the first study to use a sham-controlled arm to examine the rate of adverse events during chiropractic care. It also appears to be the first controlled study to investigate how often adverse events occur during chiropractic care for spinal pain, as the only other controlled chiropractic study examined adverse events for chiropractic treatment of neck pain. The findings of this study will build on the limited number of previous studies conducted to examine adverse events during chiropractic care and contribute towards a more robust understanding of the safety profile of chiropractic care. In particular, the inclusion of a sham arm will enable us to better discern the rate of actual adverse events by comparing the frequency of adverse events that occurred in the usual chiropractic care group to those in the sham controlled group. This study will also provide additional evidence about the efficacy of short term usual chiropractic care for spinal pain.

There are three main limitations associated with the conduct of this study. First, we will only be able to provide two treatment sessions to each participant due to funding constraints. Consequently, in all likelihood our study will only capture the most common adverse events of chiropractic care. Second, the internal validity of this trial would have been enhanced by having the same chiropractors provide both interventions. However, we decided to use a different group of chiropractors to provide each intervention as we anticipated difficulty in recruiting sufficient chiropractors with access to both ultrasound and Activator^® ^instruments, both of which are required to administer the sham intervention. Third, the extent to which we can accurately determine the relative risk of usual chiropractic care depends on how well we are able to maintain blinding to treatment allocation. We acknowledge that it may be difficult to blind the participants with spinal pain to the assigned treatment throughout the trial and accordingly will examine blinding through administering a questionnaire. However, our findings about the success of blinding should be viewed cautiously as the questionnaire we will use to assess blinding has not been formally validated.

## Abbreviations

BI: Bang Index; CONSORT: Consolidated Standards of Reporting Trials; FABQ: Fear-Avoidance Behaviour Questionnaire; FRI: Functional Rating Index; NDI: Neck Disability Index; NRS: Numerical Rating Scale; ODI: Oswestry Disability Index; PCS: Pain Catastrophising Scale; TENS: Transcutaneous Nerve Stimulation.

## Competing interests

The authors declare that they have no competing interests.

## Authors' contributions

BFW was involved in the conceptualisation of the project, the design and drafting the manuscript. JH was involved in the conceptualisation of the project, the design and drafting the manuscript. SF was involved in the conceptualisation of the project, the design and drafting the manuscript. BRC was involved with the design and statistics and manuscript drafting. BL was involved in the conceptualisation of the project and the design. NJS was involved in the design and drafting of the manuscript. All authors have read and approved the final version of the manuscript.

## Supplementary Material

Additional File 1 **Adverse Events in Chiropractic Care Questionnaire**. Survey instrument developed for this study to gather information about adverse events. Click here for file

## References

[B1] Walker BF French SD Grant W Green S Combined Chiropractic Interventions for Low Back Pain Cochrane Database Syst Rev 2010 4 CD005427 20393942 10.1002/14651858.CD005427.pub2 PMC6984631

[B2] Rubinstein SM van Middelkoop M Assendelft WJ de Boer MR van Tulder MW Spinal manipulative therapy for chronic low-back pain Cochrane Database Syst Rev 2011 2 CD008112 21328304 10.1002/14651858.CD008112.pub2 PMC12009663

[B3] Senstad O Leboeuf-Yde C Borchgrevink C Frequency and Characteristics of Side Effects of Spinal Manipulative Therapy Spine 1996 22 435 440 10.1097/00007632-199702150-00017 9055373

[B4] Hurwitz EL Morgenstern H Vassilaki M Chiang LM Adverse Reactions to Chiropractic Treatment and Their Effects on Satisfaction and Clinical Outcomes among Patients Enrolled In UCLA Neck Pain Study J Manipulative Physiol Ther 2004 27 16 25 10.1016/j.jmpt.2003.11.002 14739870

[B5] Thiel HW Bolton JE Docherty S Portlock JC Safety of Chiropractic Manipulation of the Cervical Spine: A Prospective National Survey Spine 2007 32 21 2375 2378 10.1097/BRS.0b013e3181557bb1 17906581

[B6] Rubinstein SM Leboeuf-Yde C Knol DL de Koekkoek TE Pfeifle CE van Tulder MW Predictors of Adverse Events Following Chiropractic Care for Patients with Neck Pain J Manipulative Physiol Ther 2008 31 2 94 103 10.1016/j.jmpt.2007.12.006 18328935

[B7] Thomson R Side Effects and Placebo amplification Br J Psychiatry 1982 1 64 68 10.1192/bjp.140.1.64 7037102

[B8] Gouveia LO Castanho P Ferreira JJ Safety of chiropractic interventions: a systematic review Spine 2009 34 E405 13 10.1097/BRS.0b013e3181a16d63 19444054

[B9] Activator Methods http://activator.com/activator-methods-an-update-and-review-part-one-of-two-2/Last Accessed: 26 May 2011

[B10] Boutron I Moher D Altman DG Schulz KF Ravaud P Extending the CONSORT statement to randomized trials of nonpharmacologic treatment: explanation and elaboration Ann Intern Med 2008 148 295 309 18283207 10.7326/0003-4819-148-4-200802190-00008

[B11] Ioannidis JP Evans SJ Gøtzsche PC O'Neill RT Altman DG Schulz K Moher D Better reporting of harms in randomized trials: an extension of the CONSORT statement Ann Intern Med 2004 141 781 8 15545678 10.7326/0003-4819-141-10-200411160-00009

[B12] Katz J Melzack R Measurement of pain Surg Clin North Am 2010 79 231 52 10.1016/s0039-6109(05)70381-9 10352653

[B13] Feise R Functional Rating Index: A New Valid and Reliable Instrument to Measure the Magnitude of Clinical Change in Spinal Conditions Spine 2001 26 78 97 10.1097/00007632-200101010-00015 11148650

[B14] Carragee EJ Cheng I Minimum Acceptable Outcome after Lumbar spinal Fusion Spine J 2010 10 313 320 10.1016/j.spinee.2010.02.001 20362247

[B15] Childs JS Piva SR Responsiveness of the Numeric Pain Rating Scale in Patients with Low back Pain Spine 2005 30 1331 1334 10.1097/01.brs.0000164099.92112.29 15928561

[B16] Lehana Thabane Jinhui Ma Rong Chu Ji Cheng Afisi Ismaila Rios Lorena P Reid Robson Marroon Thaban Lora Giangregorio Goldsmith Charles H A tutorial on pilot studies: the what, why and how BMC Med Res Methodol 2010 10 1 10.1186/1471-2288-10-1 20053272 PMC2824145

[B17] Koes BW Bouter LM van Mameren H Essers AH Verstegen GJ Hofhuizen DM Houben JP Knipschild PG Randomised clinical trial of manipulative therapy and physiotherapy for persistant back and neck pain complaints: results of one year follow up BMJ 1992 304 601 605 10.1136/bmj.304.6827.601 1532760 PMC1881307

[B18] Pengel L Outcomes of recent onset of low back pain (PhD thesis) Physiotherapy 2004 Sydney, The University of Sydney

[B19] Waddell G Newton M Henderson I Somerville D Main CJ Fear Avoidance Beliefs Questionnaire (FABQ) and the role of fear-avoidance beliefs in chronic low back pain and disability Pain 1993 52 157 168 10.1016/0304-3959(93)90127-B 8455963

[B20] Ware JE Jr Sherbourne CD The MOS 36-item short-form health survey (SF-36). Conceptual framework and item selection Med Care 1992 30 473 483 10.1097/00005650-199206000-00002 1593914

[B21] Hill JC Dunn KM Lewis M Mullis R Main CJ Foster NE Hay EM A primary care back pain screening tool: identifying patient subgroups for initial treatment Arthritis Rheum 2008 59:5 632 41 10.1002/art.23563 18438893

[B22] Sullivan MJL Bishop SR Pivik J The pain catastrophizing scale: development and validation Psychol Assess 1995 7 524 532

[B23] Fritz J Irrgang J A comparison of a modified Oswestry Low Back Pain Disability Questionnaire and the Quebec Back Pain Disability Scale Phys Ther 2001 81 776 788 11175676 10.1093/ptj/81.2.776

[B24] Vernon H Mior S The Neck Disability Index: A study of reliability and validity J Manipulative Physiol Ther 1991 14 409 15 1834753

[B25] Garratt AM Ruta DA Abdalla MI Buckingham JK Russell IT The SF36 health survey questionnaire: an outcome measure suitable for routine use within the NHS? BMJ 306 1440 1444 10.1136/bmj.306.6890.1440 PMC1677883 8518640

[B26] Australian Bureau of Statistics 1291.0 - A Guide to Major ABS Classifications http://www.abs.gov.au/ausstats/abs@.nsf/DirClassManualsbyTopic/F19DB188D50D978ACA2570B30006A35D?OpenDocument Last Accessed 19 April 2011

[B27] Dworkin RH Turk DC McDermott MP Peirce-Sandner S Burke LB Cowan P Farrar JT Hertz S Raja SN Rappaport BA Rauschkolb C Sampaio C Interpreting the Clinical Importance of Treatment Outcomes in Chronic Pain Clinical Trials: IMMPACT Recommendations J Pain 2005 9 105 121 10.1016/j.jpain.2007.09.005 18055266

[B28] D'Eon JL Harris CA Ellis JA Testing factorial validity and gender invariance of the pain catastrophizing scale J Behav Med 2004 27 361 72 15559733 10.1023/b:jobm.0000042410.34535.64

[B29] Cagnie B Vinck E Beernaert A Cambier D How common are side effects of spinal manipulation and can these side effects be predicted? Man Ther 2004 9 151 156 10.1016/j.math.2004.03.001 15245709

[B30] Gemmell H Miller P Relative effectiveness and adverse effects of cervical manipulation, mobilisation and the activator instrument in patients with sub-acute non-specific neck pain: results from a stopped randomised trial Chiropr Osteopat 2010 18 20 10.1186/1746-1340-18-20 20618936 PMC2927873

[B31] Hondras MA Long CR Cao Y Rowell RM Meeker WC A Randomized Controlled Trial Comparing Two Types of Spinal Manipulation and Minimal Conservative Medical Care for Adults 55 Years and Older with Subacute or Chronic Low Back Pain J Manipulative Physiol Ther 2009 32 330 43 10.1016/j.jmpt.2009.04.012 19539115

[B32] Santilli V Beghi E Finucci S Chiropractic manipulation in the treatment of acute back pain and sciatica with disc protrusion: a randomized double-blind clinical trial of active and simulated spinal manipulations Spine J 2006 6 131 7 10.1016/j.spinee.2005.08.001 16517383

[B33] Xue CC Zhang AL Lin V Myers R Polus B Story DF Acupuncture, chiropractic and osteopathy use in Australia: a national population survey BMC Public Health 2008 8 105 10.1186/1471-2458-8-105 18377663 PMC2322980

[B34] Bent S Padula A Avins AL Brief Communication: Better ways to question patients about adverse medical events: a randomized, controlled trial Ann Intern Med 2006 144:4 257 61 10.7326/0003-4819-144-4-200602210-00007 16490911

[B35] Kamper SJ Maher CG Mackay G Global rating of change scales: a review of strengths and weaknesses and considerations for design J Man Manip Ther 2009 17 3 163 70 20046623 10.1179/jmt.2009.17.3.163 PMC2762832

[B36] Bang NL Ni L Davis CE Assessment of Blinding in Clinical Trials Control Clin Trials 2004 25 2 143 56 10.1016/j.cct.2003.10.016 15020033

[B37] Costa LO Maher CG Latimer J Hodges PW Herbert RD Refshauge KM McAuley JH Jennings MD Motor Control Exercise for Chronic Low Back Pain: A Randomized Placebo-Controlled Trial Phys Ther 2009 89 12 1275 86 10.2522/ptj.20090218 19892856

[B38] Faul F Erdfelder E Lang AG Buchner A G*Power 3: A flexible statistical power analysis for the social, behavioral, and biomedical sciences Behav Res Method 2007 39 175 191 10.3758/BF03193146 17695343

[B39] Sistrom CL Garvan CW Proportions, odds, and risk Radiology 2004 230 12 9 10.1148/radiol.2301031028 14695382

[B40] Strauss SE Glasziou P Richardson SW Haynes RB Evidence-Based Medicine: how to practice & teach EBM 2011 4 Churchill Livingstone, London

[B41] He Y Missing Data Analysis Using Multiple Imputation: Getting to the Heart of the Matter Circ Cardiovasc Qual Outcomes 2010 3 98 105 10.1161/CIRCOUTCOMES.109.875658 20123676 PMC2818781

