# Supplementary material for: Outcomes of usual chiropractic, harm & efficacy, the ouch study: study protocol for a randomized controlled trial
Source: Trials. 2011 Oct 31;12:235. doi: 10.1186/1745-6215-12-235 (PMC3224760; doi:10.1186/1745-6215-12-235)
Supplement: Additional File 1 — Adverse Events in Chiropractic Care Questionnaire. Survey instrument developed for this study to gather information about adverse events. [file 1745-6215-12-235-S1.DOC]

**Adverse Events in Chiropractic Care**

Apart from measuring any improvement of your condition we are seeking to develop an understanding of adverse events that people may experience after chiropractic treatment. Adverse events are undesirable reactions to treatment, which in this study may include reactions such as pain or muscle tightness. Your support in completing this adverse events questionnaire would be greatly appreciated as it will provide valuable information about the types of adverse events people may experience after chiropractic care. We estimate that this questionnaire will take 10-15 minutes to complete. Thank you for taking the time to complete this questionnaire.

**Part A**

**Question 1. Did you experience any new unwelcome symptoms OR an increase of your presenting symptoms during the first 48 hours (two days) after treatment? This includes pain, discomfort or restrictions in daily activities (for example walking, washing, or dressing)**

**❑** Yes ❑ No

If you answered **No** to **Question 1**, please stop here. If you answered **Yes** to **Question 1**, please turn the page over and continue.

**Part B**

If you experienced any complaints after chiropractic treatment, please mark the boxes and scales below to indicate how long after chiropractic treatment the complaint started, how long the complaint lasted, and how severe the complaint was.

**Did you feel any muscle stiffness after the chiropractic appointment?**

**❑** Yes ❑ No

If yes, please answer the next three questions.

**How long after the appointment did you notice the stiffness?**

❑ less than 10 minutes ❑ 10 minutes to 1 hour ❑ 1 to 4 hours ❑ 4 to 24 hours ❑ more than 24 hours

**How long did the stiffness last for?**

❑ less than 1 hour ❑ 1 to 12 hours ❑ 12 to 24 hours ❑ 24 to 48 hours ❑ more than two days

**Please indicate how severe the stiffness was (1-3=mild; 4-6=moderate; 7-10 severe)**

0 1 2 3 4 5 6 7 8 9 10

**Did you experience any increase in pain levels after the chiropractic appointment?**

**❑** Yes ❑ No

If yes, please answer the next three questions.

**How long after the appointment did you first notice the increase in pain?**

❑ less than 10 minutes ❑ 10 minutes to 1 hour ❑ 1 to 4 hours ❑ 4 to 24 hours ❑ more than 24 hours

**How long did the increase in pain last for?**

❑ less than 1 hour ❑ 1 to 12 hours ❑ 12 to 24 hours ❑ 24 to 48 hours ❑ more than two days

**Please indicate how severe the pain was (1-3=mild; 4-6=moderate; 7-10 severe)**

0 1 2 3 4 5 6 7 8 9 10

**Did you experience a headache after the chiropractic appointment?**

**❑** Yes ❑ No

If yes, please answer the next three questions.

**How long after the appointment did you first notice the headache?**

❑ less than 10 minutes ❑ 10 minutes to 1 hour ❑ 1 to 4 hours ❑ 4 to 24 hours ❑ more than 24 hours

**How long did the headache last for?**

❑ less than 1 hour ❑ 1 to 12 hours ❑ 12 to 24 hours ❑ 24 to 48 hours ❑ more than two days

**Please indicate how severe the headache was (1-3=mild; 4-6=moderate; 7-10 severe)**

0 1 2 3 4 5 6 7 8 9 10

**Did you experience any radiating discomfort (numbness, tingling, nerve like sensations) after the chiropractic appointment?**

**❑** Yes ❑ No

If yes, please answer the next three questions.

**How long after the appointment did you first notice the radiating discomfort?**

❑ less than 10 minutes ❑ 10 minutes to 1 hour ❑ 1 to 4 hours ❑ 4 to 24 hours ❑ more than 24 hours

**How long did the radiating discomfort last for?**

❑ less than 1 hour ❑ 1 to 12 hours ❑ 12 to 24 hours ❑ 24 to 48 hours ❑ more than two days

**Please indicate how severe the radiating discomfort was (1-3=mild; 4-6=moderate; 7-10 severe)**

0 1 2 3 4 5 6 7 8 9 10

**Did you experience any other unwelcome symptoms? If yes please list on the line below**

**How long after the appointment did you first notice this unwelcome symptom?**

❑ less than 10 minutes ❑ 10 minutes to 1 hour ❑ 1 to 4 hours ❑ 4 to 24 hours ❑ more than 24 hours

**How long did the unwelcome symptom last for?**

❑ less than 1 hour ❑ 1 to 12 hours ❑ 12 to 24 hours ❑ 24 to 48 hours ❑ more than two days

**Please indicate how severe this unwelcome symptom was (1-3=mild; 4-6=moderate; 7-10 severe)**

0 1 2 3 4 5 6 7 8 9 10

**Did you experience any other unwelcome symptoms? If yes please list on the line below**

**How long after the appointment did you first notice this unwelcome symptom?**

❑ less than 10 minutes ❑ 10 minutes to 1 hour ❑ 1 to 4 hours ❑ 4 to 24 hours ❑ more than 24 hours

**How long did this unwelcome symptom last for?**

❑ less than 1 hour ❑ 1 to 12 hours ❑ 12 to 24 hours ❑ 24 to 48 hours ❑ more than two days

**Please indicate how severe this unwelcome symptom was (1-3=mild; 4-6=moderate; 7-10 severe)**

0 1 2 3 4 5 6 7 8 9 10

**Did you experience any other unwelcome symptoms? If yes please list on the line below**

**How long after the appointment did you first notice this unwelcome symptom?**

❑ less than 10 minutes ❑ 10 minutes to 1 hour ❑ 1 to 4 hours ❑ 4 to 24 hours ❑ more than 24 hours

**How long did this unwelcome symptom last for?**

❑ less than 1 hour ❑ 1 to 12 hours ❑ 12 to 24 hours ❑ 24 to 48 hours ❑ more than two days

**Please indicate how severe this unwelcome symptom was (1-3=mild; 4-6=moderate; 7-10 severe)**

0 1 2 3 4 5 6 7 8 9 10

**Did you experience any other unwelcome symptoms? If yes please list on the line below**

**How long after the appointment did you first notice this unwelcome symptom?**

❑ less than 10 minutes ❑ 10 minutes to 1 hour ❑ 1 to 4 hours ❑ 4 to 24 hours ❑ more than 24 hours

**How long did the unwelcome symptom last for?**

❑ less than 1 hour ❑ 1 to 12 hours ❑ 12 to 24 hours ❑ 24 to 48 hours ❑ more than two days

**Please indicate how severe this unwelcome symptom was (1-3=mild; 4-6=moderate; 7-10 severe)**

0 1 2 3 4 5 6 7 8 9 10

**Part C**

For the following questions, please indicate which, if any, problems you had with the activities of daily living after your chiropractic treatment.

**Have you had problems with sitting up in bed, or getting up from bed, or changing positions in bed?**

**❑** Yes ❑ No

**Have you had problems moving from one seat to another seat, or problems moving from sitting to standing, or problems getting into bed?**

**❑** Yes ❑ No

**Have you had problems with walking or problems with climbing steps or problems coming down steps?**

**❑** Yes ❑ No

**Have you had problems with dressing like putting on shoes or socks, or problems with putting clothes on either the upper body or lower body?**

**❑** Yes ❑ No

**Have you had any problems with personal hygiene like washing or grooming?**

**❑** Yes ❑ No

**Have you had any problems with eating or drinking?**

**❑** Yes ❑ No

**Were there any other problems you experienced after the chiropractic appointment? Please list on the space provided below**

**Have you had any type of care for your complaints during the last four weeks?**

**❑** Yes ❑ No

**Thank you for taking the time to complete this questionnaire**
